# Scalable biological-cognitive profiling for Alzheimer’s disease in the population

**DOI:** 10.1093/braincomms/fcag168

**Published:** 2026-06-01

**Authors:** Karin Lohi, Aino Aaltonen, Sanna-Kaisa Herukka, Tarja Kokkola, Sari Kärkkäinen, Mia Urjansson, FinnGen, Aarno Palotie, Mark Daly, Bridget Riley-Gills, Howard Jacob, Coralie Viollet, Slavé Petrovski, Alix Berton, Santha Ramakrishnan, Ellen Tsai, Zhihao Ding, Emily Holzinger, Robert Plenge, Joseph Maranville, Mark McCarthy, Rion Pendergrass, Jonathan Davitte, Chia-Yen Chen, Melis Atalar Aksit, Anna Vlahiotis, Katherine Klinger, Clement Chatelain, Jorg Blankenstein, Karol Estrada, Robert Graham, Dawn Waterworth, Chris O´Donnell, Nicole Renaud, Tomi P Mäkelä, Jaakko Kaprio, Minna Ruddock, Lila Kallio, Antti Hakanen, Terhi Kilpi, Markus Perola, Jukka Partanen, Taneli Raivio, Eero Punkka, Teija Kekonen, Raisa Serpi, Kati Kristiansson, Sanna Siltanen, Veli-Matti Kosma, Arto Mannermaa, Jari Laukkanen, Tiina Jokela, Mervi Ahlroth, Johanna Mäkelä, Outi Tuovila, Jeffrey Waring, Bridget Riley-Gillis, Fedik Rahimov, Ioanna Tachmazidou, Slavé Petrovski, Alix Berton, Santha Ramakrishnan, Ellen Tsai, Zhihao Ding, Marc Jung, Hanati Tuoken, Shameek Biswas, Benjamin Sun, Rion Pendergrass, Jonathan Davitte, Neha Raghavan, Jae-Hoon Sul, Melis Atalar Aksit, Xinli Hu, Katherine Klinger, Robert Graham, Dawn Waterworth, Nicole Renaud, Ma´en Obeidat, Jonathan Chung, Jonas Zierer, Mari Niemi, Samuli Ripatti, Johanna Schleutker, Markus Perola, Tiina Wahlfors, Mikko Arvas, Olli Carpén, Reetta Hinttala, Johannes Kettunen, Arto Mannermaa, Katriina Aalto-Setälä, Mika Kähönen, Jari Laukkanen, Johanna Mäkelä, Hanna Kujala, Triin Laisk, Natalia Pujol, Mika Kähönen, Veikko Salomaa, Jaana Suvisaari, Satu Koskela, Jouni Lauronen, Kristiina Aittomäki, Pirkko Pussinen, Tuomo Meretoja, Heikki Joensuu, Peeter Karihtala, Emma Juuri, Aino Salminen, Tuula Salo, David Rice, Pekka Nieminen, Ulla Palotie, Fredrik Åberg, Daniel Gordin, Patrik Finne, Joni A Turunen, Minna Raivio, Pentti Tienari, Martti Färkkilä, Jukka Koskela, Sampsa Pikkarainen, Kari Eklund, Paula Kauppi, Daniel Gordin, Juha Sinisalo, Marja-Riitta Taskinen, Tiinamaija Tuomi, Timo Hiltunen, Johanna Mattson, Eveliina Salminen, Terhi Ollila, Katariina Hannula-Jouppi, Oskari Heikinheimo, Ilkka Kalliala, Lauri Aaltonen, Erkki Isometsä, Antti Aarnisalo, Ilkka Immonen, Salla Ranta, Filip Scheperjans, Felix Vaura, Nina Mars, Esa Pitkänen, Hannele Laivuori, Tuomo Kiiskinen, Katja Kivinen, Elisabeth Widen, Taru Tukiainen, Hanna Ollila, Elmo Saarentaus, Anne Kerola, Eero Vuoksimaa, Joni Lindbohm, Zhiyu Yang, Matthew Sampson, Michelle McNulty, Aoxing Liu, Joel Rämö, Austin Argentieri, Amanda Elliott, Elisa Rahikkala, Kirsi Sipilä, Valtteri Julkunen, Ville Leinonen, Sanna Toppila-Salmi, Mikko Hiltunen, Eino Solje, Hannu Kankaanranta, Antti Mäkitie, Iiris Hovatta, Niko Välimäki, Minttu Marttila, Anne Portaankorva, Eija Laakkonen, Heidi Silven, Eeva Sliz, Riikka Arffman, Susanna Savukoski, Riitta Kaarteenaho, Jaakko Tyrmi, Laura Kuusalo, Laura Pirilä, Tapio Hellman, Matti Vuori, Teemu Niiranen, Timo Blomster, Johanna Huhtakangas, Terttu Harju, Kaisa Tasanen, Laura Huilaja, Vuokko Anttonen, Marja Vääräsmäki, Outi Uimari, Laure Morin-Papunen, Maarit Niinimäki, Terhi Piltonen, Reetta Kälviäinen, Valtteri Julkunen, Hilkka Soininen, Mikko Kiviniemi, Oili Kaipiainen-Seppänen, Margit Pelkonen, Päivi Auvinen, Maria Siponen, Liisa Suominen, Päivi Mäntylä, Kai Kaarniranta, Jukka Peltola, Airi Jussila, Katri Kaukinen, Pia Isomäki, Jussi Hernesniemi, Annika Auranen, Hannu Uusitalo, Teea Salmi, Venla Kurra, Laura Kotaniemi-Talonen, Argyro Bizaki-Vallaskangas, Juha Rinne, Roosa Kallionpää, Markku Voutilainen, Antti Palomäki, Laura Pirilä, Riitta Lahesmaa, Kaj Metsärinne, Jenni Aittokallio, Klaus Elenius, Sirkku Peltonen, Leena Koulu, Ulvi Gursoy, Varpu Jokimaa, Tytti Willberg, Adam Ziemann, Nizar Smaoui, Anne Lehtonen, Apinya Lertratanakul, Relja Popovic, Mengzhen Liu, Anneke Den Hollander, Jan Freudenberg, Britney Milkovich, Andrew Blumenfeld, Tushar Kumar, Dirk Paul, Bram Prins, Eleanor Wheeler, Kousik Kundu, Santosh Atanur, Andrew Lowe, Thomas Spargo, Oliver Burren, Margarete Fabre, Fabio Baschiera, Hans van Leeuwen, Himanshu Manchanda, Karl Heilbron, Martin Rao, Nicole Schmidt, Samu Kurki, Johanna Mielke, Juho Immonen, Thomas Battram, Tobias Hogrebe, Susan Eaton, Ketian Yu, Stephanie Loomis, Coro Paisan-Ruiz, Elke Markert, Frank Li, Yao Hu, Christoph Ogris, Eric Simon, Julio Cesar Bolivar Lopez, Monika Frysz, Marla Hochfeld, Cara Carty, Michael Turchin, Neelakshi Jog, Corneliu Bodea, Janie Shelton, Chen Li, Kritika Singh, Peng Jiang, Stephanie Loomis, Elena Sanchez, Lilith Moss, Zijie Zhao, Anna Podgornaia, Natalie Bowers, Edmond Teng, Tim Lu, Hubert Chen, Jennifer Schutzman, Erich Strauss, Hao Chen, David Choy, Rion Pendergrass, Brian Yaspan, Cameron Adams, Mark McCarthy, Michael Rothenberg, Rion Pendergrass, Sergio Dellepiane, Anubha Mahajan, Michael Holmes, Anubha Mahajan, Diana Chang, Tushar Bhangale, Fanli Xu, Laura Addis, John Eicher, Linda McCarthy, Jorge Esparza Gordillo, Joanna Betts, Rajashree Mishra, Audrey Chu, Diptee Kulkarni, Janet Kumar, Charli Harlow, Lea Sarow-Blat, Diana L Cousminer, Jagtar Nijjar, Jessica Chao, Michal Magid, Shashank Jariwala, Chris Floyd, Dan Swerdlow, Erding Hu, Prerak Desai, Stephen Haddad, Damien Croteau-Chonka, Billy Fahy, Paola Bronson, Kirsi Auro, David Pulford, Sauli Vuoti, Dermot Reilly, Karen He, Ekaterina Khramtsova, Amy Hart, Meijian Guan, Alessandro Porello, P Dunnmon, Sara Gale, Brice Keyes, John Kwon, Jonathan Sherlock, Matt Loza, Chris Whelan, W Galpern, Yanfei Zhang, Mona Selej, Abolfazl Doostparast Torshizi, Qingqin S Li, Sahar Mozzafari, Christopher Deboever, Jason Miller, Fabiana Farias, Andrey Loboda, Jorge Del-aguila, Elisabeth Vollmann, Jozsef Karman, Julie Fiore, Rajesh Kamath, Andrei Popescu, Delphine Fagegaltier, Travis Barr, Aristide Merola, Oliver Freeman, Simonne Longerich, Enrico Ferrero, Nikos Patsopoulos, Nancy Finkel, Sabina Pfister, Shola Richards, Katherine Mccauley, Xiaobo Xia, Mike Mendelson, Majd Mouded, Debby Ngo, Kirsi Kalpala, Melissa Miller, Nan Bing, Jaakko Parkkinen, Heli Lehtonen, Stefan McDonough, Ying Wu, Erin Macdonald-Dunlop, Jessica Chung, Michael McLean, Joshua Chiou, Hye In Kim, Sivakumar Pitchumani, Sumedha Jassal, Madhurima Saxena, Catherine O’Riordan, Samuel Lessard, Suzanne Jacobs, Hamid Mattoo, David Habiel, Guanling Huan, Lila Kallio, Tiina Wahlfors, Jukka Partanen, Eero Punkka, Raisa Serpi, Sanna Siltanen, Veli-Matti Kosma, Tiina Jokela, Anu Jalanko, Risto Kajanne, Mervi Aavikko, Helen Cooper, Denise Öller, Tarja Laitinen, Rodos Rodosthenous, Sofia Kuitunen, Mitja Kurki, Juha Karjalainen, Pietro Della Briotta Parolo, Arto Lehisto, Juha Mehtonen, Reza Jabal, Mutaamba Maasha, Sanni Ruotsalainen, Samuel Jones, Raymond Walters, Paavo Häppölä, Topi Paavilainen, L Elisa Lahtela, Johanna Paltta, Juulia Partanen, Olli K Pietiläinen, Veera Timonen, Mari Kaunisto, Elina Kilpeläinen, Tianduanyi Wang, Timo P Sipilä, Oluwaseun Alexander Dada, Awaisa Ghazal, Rigbe Weldatsadik, Jaska Uimonen, Kati Donner, Anu Loukola, Päivi Laiho, Susanna Lemmelä, Teemu Paajanen, Arto Pietilä, Aki Havulinna, Auli Toivola, Kristina Zguro, Mary Pat Reeve, Shanmukha Sampath Padmanabhuni, Harri Siirtola, Javier Gracia-Tabuenca, Marika Kaakinen, Shuang Luo, Vincent Llorens, Dawit Yohannes, Iina Laak, Mervi Ahlroth, Johanna Mäkelä, Pauli Wihuri, Tom Southerington, Meri Lähteenmäki, Aarno Palotie, Heiko Runz, Jaakko Kaprio, Valtteri Julkunen, Toni Saari, Eero Vuoksimaa

**Affiliations:** Institute for Molecular Medicine Finland (FIMM), HiLIFE, University of Helsinki, 00290 Helsinki, Finland; Institute for Molecular Medicine Finland (FIMM), HiLIFE, University of Helsinki, 00290 Helsinki, Finland; Department of Neurology, Institute of Clinical Medicine, University of Eastern Finland, 70210 Kuopio, Finland; Department of Neurology, NeuroCenter, Kuopio University Hospital, 70210 Kuopio, Finland; Department of Neurology, Institute of Clinical Medicine, University of Eastern Finland, 70210 Kuopio, Finland; Department of Neurology, Institute of Clinical Medicine, University of Eastern Finland, 70210 Kuopio, Finland; Institute for Molecular Medicine Finland (FIMM), HiLIFE, University of Helsinki, 00290 Helsinki, Finland; Institute for Molecular Medicine Finland (FIMM), HiLIFE, University of Helsinki, 00290 Helsinki, Finland; Analytic and Translational Genetics Unit, Massachusetts General Hospital, Boston, MA 02114, USA; The Stanley Center for Psychiatric Research and Program in Medical and Population Genetics, The Broad Institute of MIT and Harvard, Cambridge, MA 02142, USA; Institute for Molecular Medicine Finland (FIMM), HiLIFE, University of Helsinki, 00290 Helsinki, Finland; European Molecular Biological Laboratories (EMBL), 69117 Heidelberg, Germany; Institute for Molecular Medicine Finland (FIMM), HiLIFE, University of Helsinki, 00290 Helsinki, Finland; Department of Neurology, Institute of Clinical Medicine, University of Eastern Finland, 70210 Kuopio, Finland; Department of Neurology, NeuroCenter, Kuopio University Hospital, 70210 Kuopio, Finland; Institute for Molecular Medicine Finland (FIMM), HiLIFE, University of Helsinki, 00290 Helsinki, Finland; Institute for Molecular Medicine Finland (FIMM), HiLIFE, University of Helsinki, 00290 Helsinki, Finland

**Keywords:** Alzheimer’s disease, episodic memory, diagnosis, plasma biomarker, semantic fluency

## Abstract

Plasma phosphorylated tau217 has been suggested as a core biomarker for establishing a biological Alzheimer’s disease diagnosis. This blood biomarker has not been studied together with scalable cognitive assessment tools in population-based samples. We investigated the prevalence of cognitive and Alzheimer’s disease biomarker abnormalities and associations between plasma phosphorylated tau217 and remotely measured cognitive function in individuals without dementia.

We used a population-based cross-sectional sample of 65–85-year-olds (*n* = 691, 57% females), excluding those with previously diagnosed Alzheimer’s disease or other dementia-causing neurodegenerative disease. Cognition was measured with a telephone-administered word list recall task (episodic memory) and animal naming (semantic fluency). Plasma phosphorylated tau217 was determined with the ALZpath assay.

The prevalence of individuals with abnormalities in tests measuring episodic memory, semantic fluency, and plasma phosphorylated tau217 was 10–13%. Higher plasma phosphorylated tau217 levels were associated with lower scores on telephone-administered cognitive tests.

We found a substantial minority of a population-based sample of individuals without a clinical diagnosis of Alzheimer’s disease to have cognitive and plasma phosphorylated tau217 profiles suggesting underlying Alzheimer’s disease. Combining plasma phosphorylated tau217 with remote cognitive assessment could be a scalable, accessible, and cost-effective protocol for screening individuals with undiagnosed or at risk for Alzheimer’s disease.

## Teaser

Lohi *et al*. examined the combination of plasma p-tau217 and telephone-administered cognitive assessment as a scalable approach for early detection of Alzheimer’s disease. About 10–13% of a population-based sample had a biological-cognitive profile (abnormal p-tau217 and cognitive impairment) consistent with Alzheimer’s disease, despite none of them having a clinical diagnosis.

## Graphical Abstract

**Graphical Abstract:**
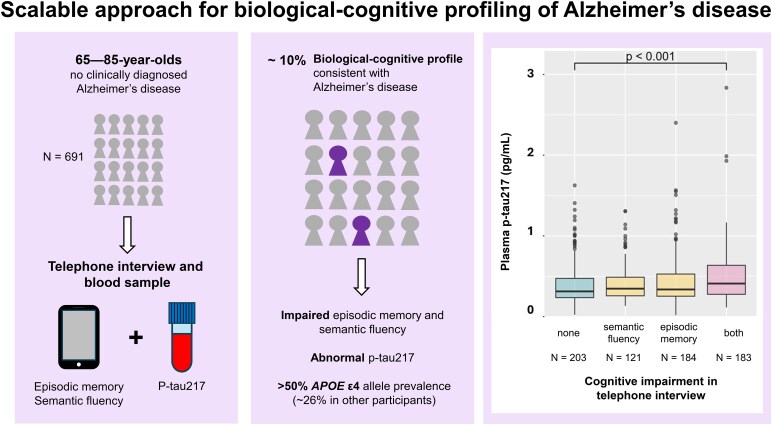

## Introduction

The current clinical practice often results in late detection of Alzheimer’s disease (AD), with a substantial proportion of patients diagnosed at the moderate or severe stage.^1^ Early diagnosis would benefit patients because current treatments are efficient in the initial stages of the disease,^2^ therefore reducing healthcare costs considerably through better disease management.^3^ Moreover, improved screening of early AD would bring an advantage to drug and intervention trials. Developing a scalable and cost-effective approach to the early detection, diagnosis, and monitoring of AD is necessary.

Plasma phosphorylated tau217 (p-tau217)—reflecting both amyloid beta (Aβ) and tau pathologies, indicating Alzheimer’s disease neuropathological changes (ADNPC)—has been suggested as a promising early biomarker to support the AD diagnostics.^4-8^ However, the AD diagnosis should not be based solely on biomarkers, and the biomarker status should not be determined in cognitively healthy individuals, as many of them are unlikely to develop symptoms in the next couple of years.^9^ In reality, many individuals who are considered ‘cognitively healthy’ may have mild or even substantial cognitive impairment simply for not having been clinically assessed.

The major advantages of plasma biomarkers over cerebrospinal fluid (CSF) samples and PET imaging are accessibility, cost-effectiveness, and applicability for large-scale use; however, research in population-based samples of cognitively normal individuals remains limited.^10^ Remote cognitive assessment with unsupervised digital tools, together with p-tau217, has been found to have prognostic value,^11^ but is not necessarily scalable at the population level, as it requires access to and knowledge of appropriate software. A telephone interview would be a more practical alternative for remote cognitive testing, particularly in low- and middle-income countries. Episodic memory and semantic fluency reflect early AD-related cognitive decline and predict progression in the AD continuum.^12^ These two cognitive domains can also be assessed remotely via a telephone interview,^13,14^ but to our knowledge, they have not been used together with plasma p-tau217 previously.

By combining a cognitive telephone interview and blood sampling, this study aimed to examine the prevalence of abnormal p-tau217 and cognitive impairment using low-cost, scalable methods in a population-based sample of individuals without a clinical diagnosis of AD or other neurodegenerative diseases. We also examined the continuous associations between telephone-administered cognitive tests and plasma p-tau217.

## Materials and methods

### Participants

Participants were 65–85-year-olds from a cross-sectional, population-based biobank recall study called TWINGEN, with data collection conducted in 2023.^15^ TWINGEN excluded individuals with AD, neurodegenerative, or other cognition-affecting disease based on health registry data up to the end of 2022. Eligibility was verified by telephone at the time of recruitment in spring 2023.^15^ We studied 691 of the total 704 TWINGEN participants who provided blood samples and participated in the telephone interview assessing cognitive performance (Supplementary Fig. 1). The TWINGEN study has ethical approval from the Helsinki and Uusimaa Hospital District (HUS) Regional Committee on Medical Research Ethics (approval number 16831/2022). In addition, permission to recontact sample donors via the Finnish Institute for Health and Welfare (THL) Biobank was granted (diary ID 83/2022). All participants provided written informed consent.

### Plasma p-tau217

Plasma was extracted from non-fasting blood samples, and aliquots were sent to the Biomarker Laboratory of the University of Eastern Finland for quantification of p-tau217 with ALZpath Simoa pTau-217 v2 Assay Kit (Quanterix, Ref# 104371).^16^ We used a Box-Cox-transformed^17^ (λ = 0.1) continuous plasma p-tau217 measure and categories based on two validated cut-offs: (>0.42 pg/mL)^16^ and (>0.475 pg/mL)^18^ to indicate dichotomic Aβ positivity (ADNPC that implies biologically defined AD). Additionally, we used a three-category ADNPC classification of plasma p-tau217 levels—low, intermediate, and high—based on cut-offs from Ashton *et al*.^16^ (<0.40, 0.40–0.63, > 0.63 pg/mL) and Figdore *et al*.^18^ (<0.405, 0.405–0.590, >0.590 pg/mL).

### Cognitive measures

We used a telephone-administered interview to assess cognition. Episodic memory was measured with a validated three-trial 10-word list-learning task using immediate (total number of words in trials 1–3; score 0–30) and delayed recall (number of words in free delayed recall; score 0–10).^19^ Semantic fluency was measured by the number of animals named in one minute.^19^

Cut-offs for cognitive impairment in our novel episodic memory measures were validated against in-person word list measures.^20^ The validity of telephone-administered semantic fluency has been demonstrated elsewhere,^21^ and the cut-off for semantic fluency impairment was based on the Finnish education-adjusted norms established for the Consortium to Establish a Registry for Alzheimer’s Disease neuropsychological battery (CERAD-nb).^22^ Furthermore, we used means (immediate recall = 14.5, delayed recall = 3.8, and semantic fluency = 15.1) from an independent sample of individuals with AD^19^ as a reference for comparing participant performance with typical AD performance, and these means are later referred to as AD scores. In addition to categorical cognitive status, we investigated continuous associations between p-tau217 and cognition. Finally, we selected delayed recall and semantic fluency to classify individuals based on the number of tests with impaired scores (i.e. impairment in zero, one, or two of these measures), given significant associations with p-tau in prior analyses.

### Statistical analysis

Descriptive statistics and sex differences were reported with Chi-squared and two-tailed T-tests. The prevalence of cognitive impairment was assessed using Chi-squared tests across dichotomic (normal/abnormal) and three-category (low/intermediate/high) p-tau217 groups. Differences in continuous plasma p-tau217 were examined with the Wald test. Benjamini-Hochberg procedure was applied to all three-category p-tau217 score and prevalence post-hoc tests.

Associations between p-tau217 and cognitive tests were analysed through Pearson correlations. Linear regression was used to examine the associations of age (centred on the sample mean), standardized plasma p-tau217 (Box-Cox transformed), and age by p-tau217 interaction with cognitive performance.

Statistical significance was considered as *P* < 0.05. All analyses accounted for participants’ relatedness (twins within families) by using the *survey* R package.^23^ We followed the Strengthening the Reporting of Observational Studies in Epidemiology guidelines for a cross-sectional study.

## Results

### Sample characteristics

The mean age of the participants was 76.17 (SD = 4.57) years, they had a median education of 10 years (IQR = 6), and 29% were *APOE* ε4-carriers with no sex differences (Table 1). Female participants performed better in immediate (*P* < 0.001) and delayed recall (*P* < 0.001; Table 1). The prevalence of Aβ positivity was 30% using the Figdore *et al*.^18^ and 38% with the Ashton *et al*.^16^ dichotomic cut-off, where no statistical difference by sex was observed. Based on the three-category approach, about 20% had high plasma p-tau217 and a high probability of ADNPC (Table 1).

**Table 1:** Sample characteristics in the whole sample and by sex

	Statistical Difference between Sexes				
	Whole sample (*N* = 691)	Females (*N* = 396)	Males (*N* = 295)	Statistic	*P*-value
Age, Mean (SD) Range	76.17 (4.57) 65.42–85.68	75.83 (4.68) 65.42–85.68	76.63 (4.40) 65.65–85.64	*t* = −1.88 (df = 538)	0.10
Education, Median (IQR) Range	10 (6) 6–18	10 (6) 6–18	10 (11) 6–18	W = 0.51 (df = 538)	0.61
*APOE* ε4-carriers, *N* (%)	200 (28.9)	114 (28.8)	86 (29.2)	*χ* ^2^ = 0.01 (ddf = 539)	0.93
Immediate recall, Mean (SD) Range	17.42 (4.23) 6–30	18.31 (4.18) 6–30	16.21 (3.99) 6–30	*t* = 6.31 (df = 538)	< 0.001
Delayed recall, Mean (SD) Range	4.76 (2.3) 0–10	5.17 (2.25) 0–10	4.21 (2.27) 0–10	*t* = 5.32 (df =538)	< 0.001
Semantic fluency, Mean (SD) Range	18.80 (5.17) 7–39	18.95 (4.92) 8–37	18.60 (5.49) 7–39	*t* = 0.82 (df = 538)	0.41
Plasma P-tau217, Median (IQR) Range	0.34 (0.30) 0.02–2.84	0.33 (0.25) 0.02–2.84	0.37 (0.36) 0.03–2.40	*t* = −1.75 (df = 538)	0.08
Dichotomic p-tau217 (Ashton *et al*.^16^), *N* (%)					
Normal	427 (61.7)	254 (64.1)	173 (58.6)	*χ* ^2^ = 1.95 (ddf = 539)	0.16
Abnormal	264 (38.2)	142 (35.9)	122 (41.4)		
Dichotomic p-tau217 (Figdore *et al*.^18^), *N* (%)					
Normal	483 (69.9)	288 (72.7)	195 (66.1)	*χ* ^2^ = 3.09 (ddf = 539)	0.08
Abnormal	208 (30.1)	108 (27.3)	100 (33.9)		
Three-category p-tau217 (Ashton *et al*.^16^), *N* (%)					
Low	407 (58.8)	245 (61.9)	162 (54.7)	*χ* ^2^ = 1.99 (ddf = 1077)	0.14
Intermediate	146 (21.1)	82 (20.7)	64 (21.7)		
High	138 (20.0)	69 (17.4)	69 (23.4)		
Three- category p-tau217 (Figdore *et al*.^18^), *N* (%)					
Low	411 (59.5)	247 (62.4)	164 (55.6)	*χ* ^2^ = 2.01 (ddf = 1077)	0.13
Intermediate	123 (17.8)	70 (17.7)	53 (18.0)		
High	157 (22.7)	79 (19.9)	78 (26.4)		

df = degrees of freedom, ddf = denominator degrees of freedom, p-tau217 = blood-based phosphorylated tau217, *χ*^2^ = Chi-squared test statistic, *t* = two-tailed *t*-test statistic.

### Cognitive performance by ADNPC status

Performance in delayed recall (t = 3.00, *P* = 0.003) and semantic fluency (t = 4.01, *P* < 0.001), but not in immediate recall (t = 1.99, *P* = 0.05), was significantly different between the dichotomic Ashton *et al*.^16^ ADNPC groups. Impairment in all three cognitive measures was significantly more common in those with ADNPC, and performance above the AD score was associated with normal plasma p-tau217 (Table 2). The results with the Figdore *et al*.^18^ cut-off (Supplementary Table 1) resembled those obtained with the Ashton *et al*.^16^ cut-off (Table 2).

**Table 2:** Cognitive performance by the Ashton *et al*.^16^ p-tau217 categories.

		Dichotomic Plasma P-tau217			Three-category Plasma P-tau217			
		Normal	Abnormal	Statistical Difference (statistic [df/ddf], *P*-value)	Low	Intermediate	High	Statistical Difference (statistic [ddf], *P*-value)
	***N***	427	264		407	146	138	
	**P-tau217 pg/ml, M (SD)**	0.27 (0.08)	0.73 (0.33)		0.26 (0.08)	0.49 (0.07)	0.94 (0.34)	
**Immediate Recall**	**Score, M (SD)**	17.68 (4.17)	16.98 (4.29)	*t* = 1.99 (538), *P* = 0.05	17.72 (4.21)	17.07 (4.30)	16.88 (4.16)	W = 2.39 (ddf = 537), *P* = 0.09
	**Impaired Cognition, *N* (%)**	183 (42.9)	141 (53.2)	*χ* ^2^ = 6.25 (539), *P* = 0.01	172 (42.3)	75 (51.4)	77 (55.4)	χ^2^ = 3.96 (ddf = 1077), *P* = 0.02
	**Score > AD score, *N* (%)**	322 (75.4)	179 (67.5)	*χ* ^2^ = 5.09 (539), *P* = 0.02	308 (75.7)	102 (70.0)	91 (65.5)	χ^2^ = 3.00 (ddf = 1078), *P* = 0.05
**Delayed Recall**	**Score, M (SD)**	4.98 (2.21)	4.40 (2.42)	*t* = 3.00 (538), *P* = 0.003	5.00 (2.21)	4.54 (2.25)	4.29 (2.56)	W = 4.72 (ddf = 537), *P* = 0.009
	**Impaired Cognition, *N* (%)**	210 (49.2)	158 (59.6)	*χ* ^2^ = 6.58 (539), *P* = 0.01	200 (49.1)	83 (56.8)	85 (61.2)	χ^2^ = 3.26 (ddf = 1073), *P* = 0.04
	**Score > AD score, *N* (%)**	312 (73.1)	174 (65.7)	*χ* ^2^ = 4.30 (539), *P* = 0.04	299 (73.5)	99 (67.8)	88 (63.3)	χ^2^ = 2.87 (ddf = 1077), *P* = 0.06
**Semantic Fluency**	**Score, M (SD)**	19.44 (5.19)	17.75 (4.98)	*t* = 4.01 (538), *P* < 0.001	19.52 (5.13)	17.90 (5.58)	17.58 (4.47)	W = 9.82 (ddf = 537), *P* < 0.001
	**Impaired Cognition, *N* (%)**	172 (40.3)	133 (50.2)	*χ* ^2^ = 5.86 (539), *P* = 0.02	161 (39.6)	78 (53.4)	66 (47.5)	χ^2^ = 4.27 (ddf = 1077), *P* = 0.01
	**Score > AD score, *N* (%)**	326 (76.3)	176 (66.4)	*χ* ^2^ = 5.83 (539), *P* = 0.02	314 (77.1)	92 (63.0)	96 (69.1)	χ^2^ = 2.89 (ddf = 1076), *P* = 0.06

AD score refers to the average telephone-based test performance of individuals with a clinical AD diagnosis (test score means: immediate recall 14.5, delayed recall 3.8, and semantic fluency 15.1).

Delayed recall (W = 4.72, *P* = 0.009) and semantic fluency (W = 9.82, *P* < 0.001) scores differed significantly across the three-category ADNPC groups (Table 2). Post-hoc tests yielded significant differences in delayed recall between low-high ADNPC groups (low-intermediate: t = −2.07, *P* = 0.06; low-high: t = −2.73, *P* = 0.02; intermediate-high: t = −0.86, *P* = 0.39) and in semantic fluency between low-high and low-intermediate ADNPC groups (low-intermediate: t = −3.03, *P* = 0.004; low-high: t = −3.98, *P* < 0.001; intermediate-high: t = −0.44, *P* = 0.66) by Ashton *et al*.^16^ cut-offs (Supplementary Table 2). No significant group differences were evident in immediate recall (W = 2.39, *P* = 0.09; Table 2). Cognitive impairment in all three cognitive measures was associated with higher p-tau217 levels by three-category classifications (*P* < 0.05; Table 2). The proportion of individuals with cognitive performance above the AD scores did not differ between the three-category Ashton *et al*.^16^ (immediate recall: *Χ*^2^ = 3.00, *P* = 0.05; delayed recall: Χ^2^ = 2.87, *P* = 0.06; semantic fluency: *Χ*^2^ = 2.89, *P* = 0.06; Table 2) and the Figdore *et al*.^18^ ADNPC groups (Supplementary Table 1).

### Continuous associations of plasma p-tau217 with cognition

**Figure 1:**
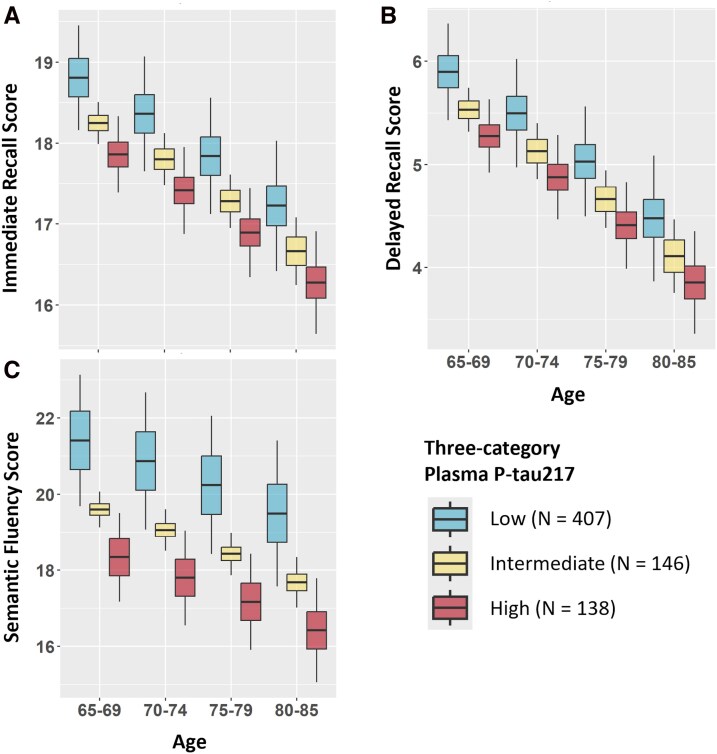
**Cognitive measures predicted by age and plasma phosphorylated tau217.** Plots display linear regression predictions of cognitive scores based on age and continuous plasma phosphorylated tau217 (p-tau217; continuous values are visualized with low, intermediate, and high groups based on the Ashton *et al*.^16^ cut-offs). Based on linear regression models, age was significantly associated with immediate recall (*t* = −2.36, *P* = 0.03; panel A), delayed recall (delayed: *t* = −4.39, *P* < 0.001; panel B), and semantic fluency (*t* = −2.46, *P* = 0.01; panel C). P-tau217 was significantly associated with semantic fluency (*t* = −3.70, *P* < 0.001; panel C), but not with immediate (*t* = −1.14, *P* = 0.26; panel A) or delayed recall (*t* = −1.45, *P* = 0.15; panel B).

Plasma p-tau217 correlated significantly with immediate recall (*r* = −0.083, *P* = 0.034), delayed recall (r = −0.113, *P* = 0.006), and semantic fluency (*r* = −0.167, *P* < 0.001; Supplementary Table 3). In linear regression analyses, p-tau217 was not a significant predictor of either immediate or delayed recall after adjusting for age (Fig. 1, Supplementary Table 4). Plasma p-tau217 was negatively associated with semantic fluency in the age-adjusted model (β = −1.251, 95% CI [−1.916, −0.587, *P* < 0.001; Fig. 1, Supplementary Table 4). Age by p-tau217 interactions were not significant for any of the cognitive measures (Supplementary Table 5).

### Plasma p-tau217 by the number of impaired cognitive scores

**Figure 2:**
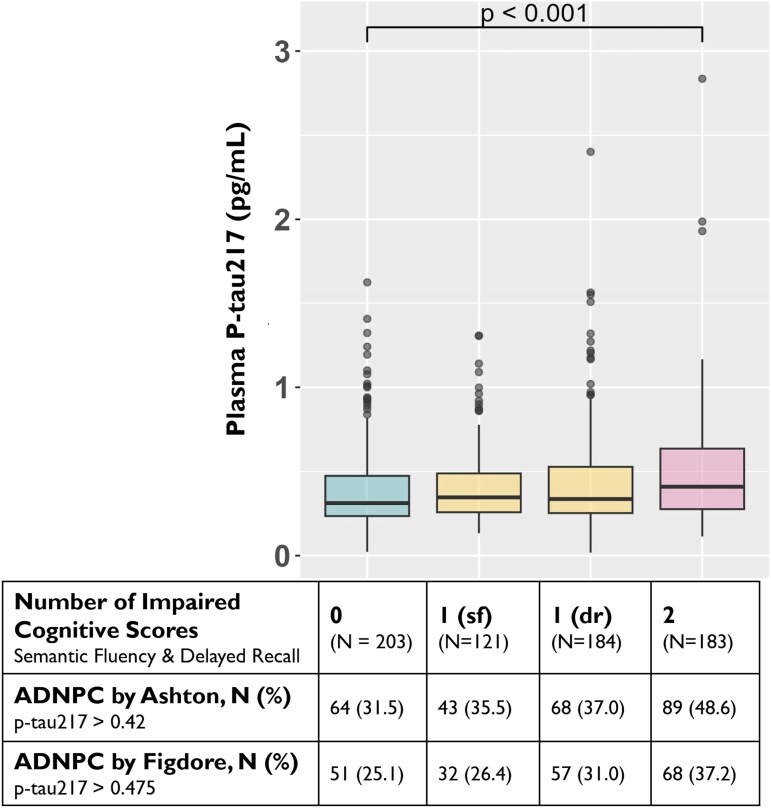
**Prevalence of Alzheimer’s disease neuropathological changes (ADNPC) by number of impaired semantic fluency (sf) and delayed recall (dr) scores.** Plasma phosphorylated tau217 (p-tau217) levels displayed by cognitive performance in two telephone-based tests: cognitively unimpaired (0), those with impairment either in semantic fluency (sf) or in delayed recall (dr), and those with impairment in both tests (2). The chi-squared test implied a significant difference in the prevalence of ADNPC between these cognitive groups based on the dichotomic Ashton *et al*.^16^ (plasma p-tau217 > 0.42), but not the Figdore *et al*.^18^ cut-off (p-tau217 > 0.475; Supplementary Table 6). In post hoc tests of continuous plasma p-tau217 levels, a significant difference was only observed between unimpaired participants and those with impairment in two tests (*t* = 3.39, *P* < 0.001) (Supplementary Tables 7–8).

Next, we used delayed and semantic fluency measures to define cognitive status by the number of impaired cognitive tests, and further investigated the association between ADNPC and cognitive status. The prevalence of ADNPC was lowest (25–32%) in participants with normative performance in both delayed recall and semantic fluency, and highest (37–49%) in participants with impaired performance in both tests (Fig. 2).

The differences in ADNPC prevalence by dichotomic p-tau217 were significantly different between cognitive status groups according to the Ashton^16^ (*χ*^2^ = 4.15, *P* = 0.006) but not according to the Figdore^18^ (*χ*^2^ = 2.47, *P* = 0.06) cut-off (Supplementary Table 6). Continuous plasma p-tau217 measures also differed significantly across cognitive status groups in the Wald-test (*W* = 3.91, *P* = 0.009). Based on post hoc tests, we found a significant difference between individuals with normal cognition and those with two impaired tests (*t* = 3.39, *P* < 0.001). In contrast, no significant differences were observed between individuals with one impaired test and those with no impairment or impairment in both tests (Fig. 2, Supplementary Tables 7–8).

The prevalence of those with biomarker and cognitive profiles consistent with AD (i.e. abnormal plasma p-tau217 and cognitive impairment on both delayed recall and semantic fluency) was 10% (*n* = 68) and 13% (*n* = 89), according to the dichotomic Figdore *et al*.^18^ and Ashton *et al*.^16^ classifications, respectively. These individuals were about 1.2–1.6 years older, based on the Figdore *et al*.^18^ (*t* = −2.31, *P* = 0.04) and the Ashton *et al*.^16^ (*t* = −3.48, *P* = 0.001) cut-offs, and had similar sex and educational distributions to those who did not fulfil these criteria (Supplementary Table 9). The AD typical biomarker-cognitive profile was also associated with a higher prevalence of *APOE* ε4-allele: 26% versus 54% (*χ*^2^ = 21.30, *P* < 0.001) according to the Figdore *et al*.^18^ cut-off and 26% versus 51% (*χ*^2^ = 21.01, *P* < 0.001) according to the Ashton *et al*.^16^ cut-off (Supplementary Table 9).

## Discussion

This study aimed to examine the prevalence of ADNPC and cognitive impairment using low-cost, scalable methods in a population-based sample without previously diagnosed dementia, and to explore the associations between cognitive performance and plasma p-tau217 in the context of advancing AD diagnosis. In this study, the telephone tests measured episodic memory and semantic fluency, cognitive domains that are considered the best predictors of progression to mild cognitive impairment and AD.^24^

Using dichotomic p-tau217, we observed a 30–38% prevalence of ADNPC in a population-based sample (mean age 76 years) and found that p-tau217 was associated with cognitive performance. In comparison, a recent study reported 34% Aβ-positivity based on CSF measures in a population-based sample (median age 63 years), where cross-sectional p-tau217 was not associated with cognition.^25^ Our results mainly aligned across continuous and categorical operationalisations of p-tau217 and cognition. Individuals with normal p-tau217 levels generally performed better in cognitive tests than those with abnormal p-tau217 levels. When using two cut-offs for p-tau217, the most consistent differences in cognitive performance were observed between the low and high p-tau217 groups, aligning with the notion that individuals with an intermediate p-tau217 level may require further diagnostic evaluation.^6^ However, when using the three-category approach, differences in immediate recall were observed only when using categorical impaired versus non-impaired classification.

Individuals with impaired performance in both delayed recall and semantic fluency had higher plasma p-tau217 levels than those who performed normally in these AD-sensitive tests. This supports the use of impairment in at least two cognitive tests as a criterion for a diagnosis of mild cognitive impairment, since an isolated abnormal score is common among cognitively healthy individuals.^26^ Importantly, we found that 10–13% of individuals without a clinical diagnosis of AD or other dementia-causing neurodegenerative disease showed concurrent episodic memory and semantic fluency impairment as measured with remote cognitive assessment, together with ADNPC determined from a blood sample. These individuals had a higher prevalence of the *APOE* ε4-allele, indicating that this scalable approach can differentiate those with a genetic risk of AD.

Despite the reported associations between p-tau217 and cognition, we note that discrepancies in AD-related biomarkers and cognitive status were common, with approximately 40% of participants exhibiting either ADNPC or impaired cognition. This aligns with earlier findings using PET and CSF measures of Aβ in those without dementia.^27,28^ In our study, the associations between continuous p-tau217 and cognitive measures were weak, highlighting the independence of ADNPC and cognition in individuals without a diagnosis of AD. The weak associations align with recent studies, suggesting that plasma p-tau217 alone or combined with *APOE* and demographic data has low accuracy for detecting cognitive impairment in those without dementia.^6,29^

Our results from population-based data are important for the ongoing discussion about the diagnosis of AD as a clinical-biological construct. The Alzheimer’s Association's workgroup guidelines included plasma p-tau217 as one of the core biomarkers, where abnormality is sufficient for diagnosing AD,^4^ while determining AD in cognitively normal individuals based on biomarkers is not recommended.^9^ However, the definition of cognitively normal in a real-world context is problematic due to the substantial prevalence of undiagnosed AD. In the clinical setting, cognitive normality is defined relative to age, sex, and education. Yet, cognitive challenges are often the reason not to seek care, leading to detrimental downstream effects for patients and clinical trials, as those with the early observations of AD benefit the most from the disease-modifying treatments.^30^

These findings indicate that combining plasma p-tau217 with telephone-administered cognitive assessment in individuals without a clinical AD diagnosis can help in classifying individuals in the AD continuum. The associations between cognitive measures and p-tau217 were weak, suggesting that cognitive measures cannot be used as proxies for ADNPC in individuals without AD. However, this approach identified individuals with both biologically defined AD and impairment in AD-sensitive cognitive domains. This finding from a real-world sample of ‘cognitively normal’ individuals suggest that using plasma p-tau217 and remote cognitive testing could improve the early detection of AD without imposing additional load on the healthcare system and would be widely accessible even without access to computers or smartphone applications.

### Strengths and limitations

Our study had several strengths. We used a population-based sample of older Finnish adults without a prior diagnosis of neurodegenerative disease, and we simultaneously analysed specific yet feasible ADNPC biomarkers and telephone-administered cognitive measures for early Alzheimer’s detection. Strength in the cognitive measures was that the episodic memory measures were validated in the TWINGEN sample, and cut-offs were determined against in-person word list measures.^31^ Similarly, the semantic fluency cut-off was based on the corresponding in-person-administered test, but validation has not been carried out in the TWINGEN sample. Other studies have indicated the validity of this semantic fluency measure administered by telephone.^20,21^

A limitation is that cognitive cut-offs were based on the Finnish education-adjusted norms for 60–81-year-olds^22^ in comparison to the age range from 65 to 85 in our sample. Using age-, sex-, and education-adjusted scores for the cognitive measures would have been ideal, but normative Finnish data for the telephone-administered measures were not yet available. Nevertheless, those who had biomarker-cognitive profiles consistent with AD did not differ from others regarding sex distribution and education level, and the differences in mean age were less than two years. This study focused on measuring episodic memory and semantic fluency, which are associated with amnestic mild cognitive impairment and risk of AD. Therefore, the results presented here may not apply to atypical variants of AD. Finally, the literature on plasma p-tau217 is rapidly growing, and several cut-offs for the ALZpath assay have been developed. With the lack of Finnish cut-offs, we used cut-offs based on two US samples,^16,18^ the latter of which had a highly similar ethnicity to our sample. Alternative cut-offs are available and, for example, Schindler *et al*.^6^ reported 0.46 pg/mL cut-off for amyloid positivity that falls in between the cut-offs used in our study.

## Conclusions

A substantial minority of older adults without diagnosed dementia had a cognitive-biological profile consistent with AD in this population-based sample. Our results indicate that detecting individuals with undiagnosed or high risk for AD is possible by combining the scalable, low-cost methods of plasma p-tau217 and remote cognitive assessment via a short telephone interview.

## Supplementary Material

fcag168_Supplementary_Data

## Data Availability

In accordance with the Finnish Biobank Act, the data used in the analysis are deposited in the Biobank of the Finnish Institute for Health and Welfare (https://thl.fi/en/our-services/thl-biobank1). It is available to researchers from academia and companies upon the submission of a written application and in accordance with relevant Finnish legislation. To ensure privacy protection and compliance with national data protection legislation, a data use/transfer agreement is needed, the content and specific clauses of which will depend on the nature of the requested data. The data analysis script is stored in a publicly available repository (https://github.com/KarinSLohi/P-tau217-x-Cognition.git).

## Appendix

FinnGen collaborators

Aarno Palotie, Mark Daly, Bridget Riley-Gills, Howard Jacob, Coralie Viollet, Slavé Petrovski, Alix Berton, Santha Ramakrishnan, Ellen Tsai, Zhihao Ding, Emily Holzinger, Robert Plenge, Joseph Maranville, Mark McCarthy, Rion Pendergrass, Jonathan Davitte, Chia-Yen Chen, Melis Atalar Aksit, Anna Vlahiotis, Katherine Klinger, Clement Chatelain, Jorg Blankenstein, Karol Estrada, Robert Graham, Dawn Waterworth, Chris O´Donnell, Nicole Renaud, Tomi P. Mäkelä, Jaakko Kaprio, Minna Ruddock, Lila Kallio, Antti Hakanen, Terhi Kilpi, Markus Perola, Jukka Partanen, Taneli Raivio, Eero Punkka, Teija Kekonen, Raisa Serpi, Kati Kristiansson, Sanna Siltanen, Veli-Matti Kosma, Arto Mannermaa, Jari Laukkanen, Tiina Jokela, Mervi Ahlroth, Johanna Mäkelä, Outi Tuovila, Jeffrey Waring, Bridget Riley-Gillis, Fedik Rahimov, Ioanna Tachmazidou, Slavé Petrovski, Alix Berton, Santha Ramakrishnan, Ellen Tsai, Zhihao Ding, Marc Jung, Hanati Tuoken, Shameek Biswas, Benjamin Sun, Rion Pendergrass, Jonathan Davitte, Neha Raghavan, Jae-Hoon Sul, Melis Atalar Aksit, Xinli Hu, Katherine Klinger, Robert Graham, Dawn Waterworth, Nicole Renaud, Ma´en Obeidat, Jonathan Chung, Jonas Zierer, Mari Niemi, Samuli Ripatti, Johanna Schleutker, Markus Perola, Tiina Wahlfors, Mikko Arvas, Olli Carpén, Reetta Hinttala, Johannes Kettunen, Arto Mannermaa, Katriina Aalto-Setälä, Mika Kähönen, Jari Laukkanen, Johanna Mäkelä, Hanna Kujala, Triin Laisk, Natalia Pujol, Mika Kähönen, Veikko Salomaa, Jaana Suvisaari, Satu Koskela, Jouni Lauronen, Kristiina Aittomäki, Pirkko Pussinen, Tuomo Meretoja, Heikki Joensuu, Peeter Karihtala, Emma Juuri, Aino Salminen, Tuula Salo, David Rice, Pekka Nieminen, Ulla Palotie, Fredrik Åberg, Daniel Gordin, Patrik Finne, Joni A Turunen, Minna Raivio, Pentti Tienari, Martti Färkkilä, Jukka Koskela, Sampsa Pikkarainen, Kari Eklund, Paula Kauppi, Daniel Gordin, Juha Sinisalo, Marja-Riitta Taskinen, Tiinamaija Tuomi, Timo Hiltunen, Johanna Mattson, Eveliina Salminen, Terhi Ollila, Katariina Hannula-Jouppi, Oskari Heikinheimo, Ilkka Kalliala, Lauri Aaltonen, Erkki Isometsä, Antti Aarnisalo, Ilkka Immonen, Salla Ranta, Filip Scheperjans, Felix Vaura, Nina Mars, Esa Pitkänen, Hannele Laivuori, Tuomo Kiiskinen, Katja Kivinen, Elisabeth Widen, Taru Tukiainen, Hanna Ollila, Elmo Saarentaus, Anne Kerola, Eero Vuoksimaa, Joni Lindbohm, Zhiyu Yang, Matthew Sampson, Michelle McNulty, Aoxing Liu, Joel Rämö, Austin Argentieri, Amanda Elliott, Elisa Rahikkala, Kirsi Sipilä, Valtteri Julkunen, Ville Leinonen, Sanna Toppila-Salmi, Mikko Hiltunen, Eino Solje, Hannu Kankaanranta, Antti Mäkitie, Iiris Hovatta, Niko Välimäki, Minttu Marttila, Anne Portaankorva, Eija Laakkonen, Heidi Silven, Eeva Sliz, Riikka Arffman, Susanna Savukoski, Riitta Kaarteenaho, Jaakko Tyrmi, Laura Kuusalo, Laura Pirilä, Tapio Hellman, Matti Vuori, Teemu Niiranen, Timo Blomster, Johanna Huhtakangas, Terttu Harju, Kaisa Tasanen, Laura Huilaja, Vuokko Anttonen, Marja Vääräsmäki, Outi Uimari, Laure Morin-Papunen, Maarit Niinimäki, Terhi Piltonen, Reetta Kälviäinen, Valtteri Julkunen, Hilkka Soininen, Mikko Kiviniemi, Oili Kaipiainen-Seppänen, Margit Pelkonen, Päivi Auvinen, Maria Siponen, Liisa Suominen, Päivi Mäntylä, Kai Kaarniranta, Jukka Peltola, Airi Jussila, Katri Kaukinen, Pia Isomäki, Jussi Hernesniemi, Annika Auranen, Hannu Uusitalo, Teea Salmi, Venla Kurra, Laura Kotaniemi-Talonen, Argyro Bizaki-Vallaskangas, Juha Rinne, Roosa Kallionpää, Markku Voutilainen, Antti Palomäki, Laura Pirilä, Riitta Lahesmaa, Kaj Metsärinne, Jenni Aittokallio, Klaus Elenius, Sirkku Peltonen, Leena Koulu, Ulvi Gursoy, Varpu Jokimaa, Tytti Willberg, Adam Ziemann, Nizar Smaoui, Anne Lehtonen, Apinya Lertratanakul, Relja Popovic, Mengzhen Liu, Anneke Den Hollander, Jan Freudenberg, Britney Milkovich, Andrew Blumenfeld, Tushar Kumar, Dirk Paul, Bram Prins, Eleanor Wheeler, Kousik Kundu, Santosh Atanur, Andrew Lowe, Thomas Spargo, Oliver Burren, Margarete Fabre, Fabio Baschiera, Hans van Leeuwen, Himanshu Manchanda, Karl Heilbron, Martin Rao, Nicole Schmidt, Samu Kurki, Johanna Mielke, Juho Immonen, Thomas Battram, Tobias Hogrebe, Susan Eaton, Ketian Yu, Stephanie Loomis, Coro Paisan-Ruiz, Elke Markert, Frank Li, Yao Hu, Christoph Ogris, Eric Simon, Julio Cesar Bolivar Lopez, Monika Frysz, Marla Hochfeld, Cara Carty, Michael Turchin, Neelakshi Jog, Corneliu Bodea, Janie Shelton, Chen Li, Kritika Singh, Peng Jiang, Stephanie Loomis, Elena Sanchez, Lilith Moss, Zijie Zhao, Anna Podgornaia, Natalie Bowers, Edmond Teng, Tim Lu, Hubert Chen, Jennifer Schutzman, Erich Strauss, Hao Chen, David Choy, Rion Pendergrass, Brian Yaspan, Cameron Adams, Mark McCarthy, Michael Rothenberg, Rion Pendergrass, Sergio Dellepiane, Anubha Mahajan, Michael Holmes, Anubha Mahajan, Diana Chang, Tushar Bhangale, Fanli Xu, Laura Addis, John Eicher, Linda McCarthy, Jorge Esparza Gordillo, Joanna Betts, Rajashree Mishra, Audrey Chu, Diptee Kulkarni, Janet Kumar, Charli Harlow, Lea Sarow-Blat, Diana L.Cousminer, Jagtar Nijjar, Jessica Chao, Michal Magid, Shashank Jariwala, Chris Floyd, Dan Swerdlow, Erding Hu, Prerak Desai, Stephen Haddad, Damien Croteau-Chonka, Billy Fahy, Paola Bronson, Kirsi Auro, David Pulford, Sauli Vuoti, Dermot Reilly, Karen He, Ekaterina Khramtsova, Amy Hart, Meijian Guan, Alessandro Porello, P. Dunnmon, Sara Gale, Brice Keyes, John Kwon, Jonathan Sherlock, Matt Loza, Chris Whelan, W Galpern, Yanfei Zhang, Mona Selej, Abolfazl Doostparast Torshizi, Qingqin S Li, Sahar Mozzafari, Christopher Deboever, Jason Miller, Fabiana Farias, Andrey Loboda, Jorge Del-aguila, Elisabeth Vollmann, Jozsef Karman, Julie Fiore, Rajesh Kamath, Andrei Popescu, Delphine Fagegaltier, Travis Barr, Aristide Merola, Oliver Freeman, Simonne Longerich, Enrico Ferrero, Nikos Patsopoulos, Nancy Finkel, Sabina Pfister, Shola Richards, Katherine Mccauley, Xiaobo Xia, Mike Mendelson, Majd Mouded, Debby Ngo, Kirsi Kalpala, Melissa Miller, Nan Bing, Jaakko Parkkinen, Heli Lehtonen, Stefan McDonough, Ying Wu, Erin Macdonald-Dunlop, Jessica Chung, Michael McLean, Joshua Chiou, Hye In Kim, Sivakumar Pitchumani, Sumedha Jassal, Madhurima Saxena, Catherine O’Riordan, Samuel Lessard, Suzanne Jacobs, Hamid Mattoo, David Habiel, Guanling Huan, Lila Kallio, Tiina Wahlfors, Jukka Partanen, Eero Punkka, Raisa Serpi, Sanna Siltanen, Veli-Matti Kosma, Tiina Jokela, Anu Jalanko, Risto Kajanne, Mervi Aavikko, Helen Cooper, Denise Öller, Tarja Laitinen, Rodos Rodosthenous, Sofia Kuitunen, Mitja Kurki, Juha Karjalainen, Pietro Della Briotta Parolo, Arto Lehisto, Juha Mehtonen, Reza Jabal, Mutaamba Maasha, Sanni Ruotsalainen, Samuel Jones, Raymond Walters, Paavo Häppölä, Topi Paavilainen, L. Elisa Lahtela, Johanna Paltta, Juulia Partanen, Olli K. Pietiläinen, Veera Timonen, Mari Kaunisto, Elina Kilpeläinen, Tianduanyi Wang, Timo P. Sipilä, Oluwaseun Alexander Dada, Awaisa Ghazal, Rigbe Weldatsadik, Jaska Uimonen, Kati Donner, Anu Loukola, Päivi Laiho, Susanna Lemmelä, Teemu Paajanen, Arto Pietilä, Aki Havulinna, Auli Toivola, Kristina Zguro, Mary Pat Reeve, Shanmukha Sampath Padmanabhuni, Harri Siirtola, Javier Gracia-Tabuenca, Marika Kaakinen, Shuang Luo, Vincent Llorens, Dawit Yohannes, Iina Laak, Mervi Ahlroth, Johanna Mäkelä, Pauli Wihuri, Tom Southerington, and Meri Lähteenmäki.

## References

[1] Alenius M, Hokkanen L, Koskinen S, et al Cognitive performance at time of AD diagnosis: A clinically augmented register-based study. Front Psychol. 2022;13:901945. 35846684 10.3389/fpsyg.2022.901945 PMC9284003

[2] Livingston G, Huntley J, Liu KY, et al Dementia prevention, intervention, and care: 2024 report of the lancet standing commission. The Lancet. 2024;404(10452):572–628. 10.1016/S0140-6736(24)01296-0 39096926

[3] 2024 Alzheimer’s disease facts and figures. Alzheimers Dement. 2024;20(5):3708–3821. 38689398 10.1002/alz.13809 PMC11095490

[4] Jack CR, Andrews SJ, Beach TG, et al Revised criteria for the diagnosis and staging of Alzheimer’s disease. Nat Med. 2024;30(8):2121–2124. 38942991 10.1038/s41591-024-02988-7 PMC11630478

[5] Jack CR, Bennett DA, Blennow K, et al NIA-AA research framework: Toward a biological definition of Alzheimer’s disease. Alzheimers Dement. 2018;14(4):535–562. 29653606 10.1016/j.jalz.2018.02.018 PMC5958625

[6] Schindler SE, Petersen KK, Saef B, et al Head-to-head comparison of leading blood tests for Alzheimer’s disease pathology. Alzheimers Dement. 2024;20(11):8074–8096. 39394841 10.1002/alz.14315 PMC11567821

[7] Brum WS, Cullen NC, Therriault J, et al A blood-based biomarker workflow for optimal tau-PET referral in memory clinic settings. Nat Commun. 2024;15(1):2311. 38486040 10.1038/s41467-024-46603-2 PMC10940585

[8] Salvadó G, Janelidze S, Bali D, et al Plasma phosphorylated tau 217 to identify preclinical Alzheimer disease. JAMA Neurol. 2025;82:1122. 40952756 10.1001/jamaneurol.2025.3217 PMC12558403

[9] Dubois B, Villain N, Schneider L, et al Alzheimer disease as a clinical-biological construct—An international working group recommendation. JAMA Neurol. 2024;81:1304. 39483064 10.1001/jamaneurol.2024.3770 PMC12010406

[10] Mielke MM, Dage JL, Frank RD, et al Performance of plasma phosphorylated tau 181 and 217 in the community. Nat Med. 2022;28(7):1398–1405. 35618838 10.1038/s41591-022-01822-2 PMC9329262

[11] Berron D, Olsson E, Andersson F, et al Remote and unsupervised digital memory assessments can reliably detect cognitive impairment in Alzheimer’s disease. Alzheimers Dement. 2024;20(7):4775–4791. 38867417 10.1002/alz.13919 PMC11247711

[12] Drago V, Babiloni C, Bartrés-Faz D, et al Disease tracking markers for Alzheimer’s disease at the prodromal (MCI) stage. J Alzheimers Dis. 2011;26(s3):159–199. 21971460 10.3233/JAD-2011-0043

[13] Järvenpää T, Rinne JO, Räihä I, et al Characteristics of two telephone screens for cognitive impairment. Dement Geriatr Cogn Disord. 2002;13(3):149–155. 11893836 10.1159/000048646

[14] Requena-Komuro MC, Jiang J, Dobson L, et al Remote versus face-to-face neuropsychological testing for dementia research: A comparative study in people with Alzheimer’s disease, frontotemporal dementia and healthy older individuals. BMJ Open. 2022;12(11):e064576. 10.1136/bmjopen-2022-064576 PMC9702828 36428012

[15] Vuoksimaa E, Saari TT, Aaltonen A, et al TWINGEN: Protocol for an observational clinical biobank recall and biomarker cohort study to identify Finnish individuals with high risk of Alzheimer’s disease. BMJ Open. 2024;14(6):e081947. 10.1136/bmjopen-2023-081947 PMC11177688 38866570

[16] Ashton NJ, Brum WS, Di Molfetta G, et al Diagnostic accuracy of a plasma phosphorylated tau 217 immunoassay for Alzheimer disease pathology. JAMA Neurol. 2024;81(3):255. 38252443 10.1001/jamaneurol.2023.5319 PMC10804282

[17] Kutner MH , ed. Applied linear statistical models. 5th ed. McGraw-Hill Irwin; 2005.

[18] Figdore DJ, Griswold M, Bornhorst JA, et al Optimizing cutpoints for clinical interpretation of brain amyloid status using plasma p-tau217 immunoassays. Alzheimers Dement. 2024;20(9):6506–6516. 39030981 10.1002/alz.14140 PMC11497693

[19] Julkunen V, Schwarz C, Kalapudas J, et al A FinnGen pilot clinical recall study for Alzheimer’s disease. Sci Rep. 2023;13(1):12641. 37537264 10.1038/s41598-023-39835-7 PMC10400697

[20] Saari TT, Aaltonen A, Lohi K, et al Validity of telephone-administered word list learning measures for assessment of episodic memory in aging and Alzheimer’s disease. Neuropsychology. 2025;40:17–35. 41196698 10.1037/neu0001019

[21] Marceaux JC, Prosje MA, McClure LA, et al Verbal fluency in a national sample: Telephone administration methods. Int J Geriatr Psychiatry. 2019;34(4):578–587. 30588700 10.1002/gps.5054 PMC6420356

[22] Hallikainen I, Alenius M, Hokkanen L, et al CERAD-tehtäväsarjaan koulutustason huomioivat katkaisurajat ja kokonaispistemäärä käyttöön. *Suom Lääkärilehti*. Published online 2023. www.laakarilehti.fi/e35809

[23] Lumley T, Gao P, Schneider B. Analysis of Complex survey samples. Version 4.4-2. CRAN; 2024. http://r-survey.r-forge.r-project.org/survey/

[24] Weissberger GH, Strong JV, Stefanidis KB, Summers MJ, Bondi MW, Stricker NH. Diagnostic accuracy of memory measures in Alzheimer’s dementia and mild cognitive impairment: A systematic review and meta-analysis. Neuropsychol Rev. 2017;27(4):354–388. 28940127 10.1007/s11065-017-9360-6 PMC5886311

[25] González-Escalante A, Milà-Alomà M, Brum WS, et al A plasma biomarker panel for detecting early amyloid-β accumulation and its changes in middle-aged cognitively unimpaired individuals at risk for Alzheimer’s disease. eBioMedicine. 2025;116:105741. 40414160 10.1016/j.ebiom.2025.105741 PMC12159936

[26] Brooks BL, Iverson GL, White T. Substantial risk of “accidental MCI” in healthy older adults: Base rates of low memory scores in neuropsychological assessment. J Int Neuropsychol Soc. 2007;13(03):490–500. 17445298 10.1017/S1355617707070531

[27] Jansen WJ, Ossenkoppele R, Knol DL, et al Prevalence of cerebral amyloid pathology in persons without dementia: A meta-analysis. JAMA. 2015;313(19):1924. 25988462 10.1001/jama.2015.4668 PMC4486209

[28] Roberts RO, Aakre JA, Kremers WK, et al Prevalence and outcomes of amyloid positivity among persons without dementia in a longitudinal, population-based setting. JAMA Neurol. 2018;75(8):970. 29710225 10.1001/jamaneurol.2018.0629 PMC6142936

[29] Warmenhoven N, Salvadó G, Janelidze S, et al A comprehensive head-to-head comparison of key plasma phosphorylated tau 217 biomarker tests. Brain. 2025;148(2):416–431. 39468767 10.1093/brain/awae346 PMC11788211

[30] Langbaum JB, Zissimopoulos J, Au R, et al Recommendations to address key recruitment challenges of Alzheimer’s disease clinical trials. Alzheimers Dement. 2023;19(2):696–707. 35946590 10.1002/alz.12737 PMC9911558

[31] Saari TT, Palviainen T, Hiltunen M, et al Cross-sectional study of plasma phosphorylated tau 217 in persons without dementia. Alzheimers Dement Diagn Assess Dis Monit. 2025;17(2):e70107. 10.1002/dad2.70107 PMC12064337 40352683

