# Supplementary material for: Scalable biological-cognitive profiling for Alzheimer’s disease in the population
Source: Brain Commun. 2026 Jun 1;8(3):fcag168. doi: 10.1093/braincomms/fcag168 (PMC13223580; doi:10.1093/braincomms/fcag168)
Supplement: fcag168_Supplementary_Data [file fcag168_supplementary_data.zip › Supplementary_material.pdf]

Supplementary Material:

Scalable biological-cognitive profiling for Alzheimer's disease in the population

Supplementary Figure 1. Flowchart of original twin participant recruitment and selection for the TWINGEN-cohort.

Finnish Twin Cohort study (FTC) was initiated in 1975, when invitation for the study was sent to all same-sex twins born before 1958. These same-sex twin pairs were followed with multiple questionnaires in 1981 and 2017. Opposite-sex twin pairs born during same decades were contacted later and received baseline questionnaire between 1995 and 1996. Biological samples of FTC participants were transferred into THL Biobank in 2018 following the Biobank Act. For TWINGEN, individuals with clinically diagnosed neurodegenerative disease or other cognition- or personality-affecting traumatic or chronic diseases were excluded from the study based on health registry data and confirmed via telephone.

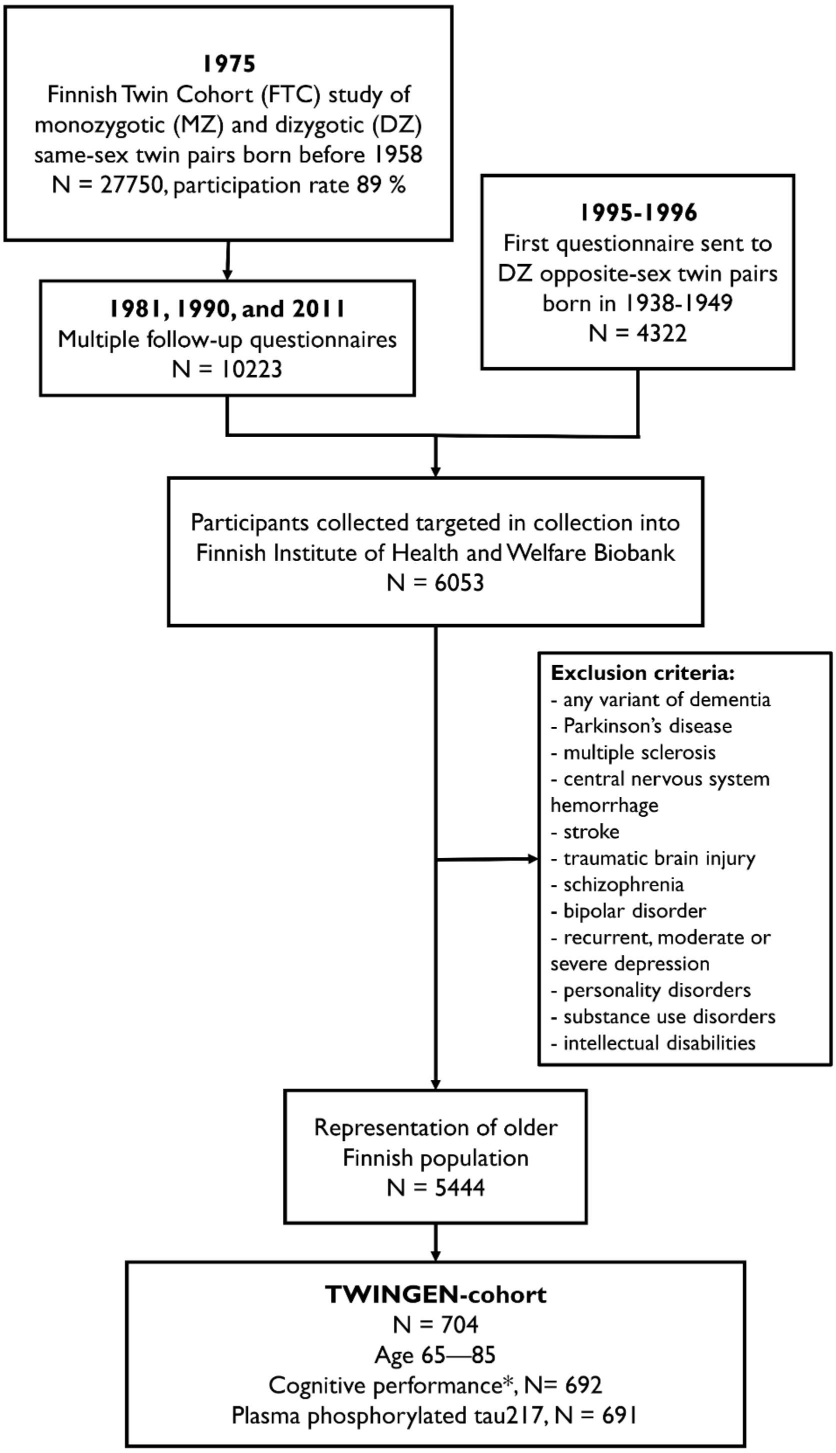

* telephone-based immediate and delayed recall, and semantic fluency scores

Supplementary Table 1: Cognitive performance variance by the Figdore et al.[1] p-tau217 categories.

		Dichotomic Plasma P-tau217			Three-category Plasma P-tau217			
		Normal	Abnormal	Statistical Difference (statistic [df[1]/ddf[2]], p-value[3])	Low	Intermediate	High	Statistical Difference (statistic [ddf], p-value[3])
	**N**	483	208		411	123	157	
	**P-tau217 pg/ml, Median (IQR)**	0.29 (0.13)	0.73 (0.32)		0.27 (0.11)	0.46 (0.07)	0.82 (0.30)	
**Immediate Recall**	**Score, M (SD)**	17.66 (4.21)	16.85 (4.22)	t = 2.25 (df = 538), p = 0.03	17.71 (4.19)	17.01 (4.28)	16.97 (4.26)	W = 2.20 (537), p = 0.11
	**Impaired Cognition, N (%)**	211 (43.7)	112 (53.8)	χ^2 = 5.66 (ddf = 539), p = 0.02	174 (42.3)	64 (52.0)	85 (54.1)	χ^2 = 3.82 (1077), p = 0.02
	**Score > AD score[4], N (%)**	326 (74.9)	138 (66.3)	χ^2 = 5,28 (ddf = 539), p = 0.02	311 (75.7)	85 (69.1)	104 (66.2)	χ^2 = 2.82 (1078), p = 0.06
**Delayed Recall**	**Score, M (SD)**	4.92 (2.22)	4.40 (2.46)	t = 2.50 (df = 538), p = 0.01	5.00 (2.20)	4.50 (2.24)	4.34 (2.54)	W = 4.63 (537), p = 0.01
	**Impaired Cognition, N (%)**	242 (50.1)	125 (60.1)	χ^2 = 5.50 (ddf = 539), p = 0.02	202 (49.1)	69 (56.1)	96 (61.1)	χ^2 = 3.47 (1072), p = 0.03
	**Score > AD score[5], N (%)**	347 (71.8)	138 (66.3)	χ^2 = 2.03 (ddf = 539), p = 0.15	302 (73.5)	82 (66.7)	101 (64.3)	χ^2 = 2.68 (1077), p = 0.07
**Semantic Fluency**	**Score, M (SD)**	19.18 (5.28)	17.91 (4.79)	t = 2.96 (df = 538), p = 0.003	19.51 (5.12)	17.99 (5.51)	17.59 (4.71)	W = 9.53 (537), p < 0.001
	**Impaired Cognition, N (%)**	204 (42.2)	100 (48.1)	χ^2 = 1.92 (ddf = 539), p = 0.17	163 (39.7)	64 (52.0)	77 (49.0)	χ^2 = 3.82 (1077), p = 0.02
	**Score > AD score[6], N (%)**	358 (74.1)	144 (69.2)	χ^2 = 4.50 (ddf = 539), p = 0.03	317 (77.1)	78 (63.4)	107 (68.2)	χ^2 = 2.97 (1078), p = 0.05

[1] Degrees of freedom
[2] Denominator degrees of freedom
[3] Benjamini-Hochberg procedure applied to all p-values.
[4] AD score refers to the average telephone-based test performance of individuals with a clinical AD diagnosis; the immediate recall average score was 14.5.
[5] AD score refers to the average telephone-based test performance of individuals with a clinical AD diagnosis; the delayed recall average score was 3.8.
[6] AD score refers to the average telephone-based test performance of individuals with a clinical AD diagnosis; the semantic fluency average score was 15.1.

Supplementary Table 2: Post-Hoc Test for cognitive performance by three-category plasma p-tau217 groups.

			**Three-category plasma p-tau217 and Cognitive Status** (statistic [DF], p-value[7])		
			**Low-Intermediate**	**Low-High**	**Intermediate-High**
**Ashton et al.**[2]	**Immediate Recall**	**Score**	t = -1.56 (441), p = 0.18	t = -1.92 (444), p = 0.17	t = -0.36 (248), p = 0.72
		**Impaired Cognition**	χ^2 = 3.44 (442), p = 0.10	χ^2 = 6.32 (445), p = 0.04	χ^2 = 0.38 (249), p = 0.54
		**Score > AD score**	χ^2 = 1.81 (442), p = 0.27	χ^2 = 5.58 (445), p = 0.06	χ^2 = 0.67 (249), p = 0.41
	**Delayed Recall**	**Score**	t = -2.07 (441), p = 0.06	t = -2.73 (444), p = 0.02	t = -0.86 (248), p = 0.39
		**Impaired Cognition**	χ^2 = 2.61 (442), p = 0.16	χ^2 = 5.16 (445), p = 0.07	χ^2 = 0.47 (249), p = 0.49
		**Score > AD score**	χ^2 = 1.70 (442), p = 0.29	χ^2 = 5.15 (445), p = 0.07	χ^2 = 0.72 (249), p = 0.40
	**Semantic Fluency**	**Score**	t = -3.03 (441), p = 0.004	t = -3.98 (444), p < 0.001	t = -0.44 (248), p = 0.66
		**Impaired Cognition**	χ^2 = 7.79 (442), p = 0.02	χ^2 = 2.30 (445), p = 0.19	χ^2 = 1.09 (249), p = 0.30
		**Score > AD score**	χ^2 = 2.94 (442), p = 0.13	χ^2 = 4.42 (445), p = 0.11	χ^2 = 0.14 (249), p = 0.71
**Figdore et al.**[1]	**Immediate Recall**	**Score**	t = -1.57 (427), p = 0.18	t = -1.77 (463), p = 0.18	t = -0.08 (245), p = 0.94
		**Impaired Cognition**	χ^2 = 3.42 (428), p = 0.10	χ^2 = 5.95 (464), p = 0.05	χ^2 = 0.12 (246), p = 0.73
		**Score > AD score**	χ^2 = 2.01 (428), p = 0.23	χ^2 = 5.05 (464), p = 0.08	χ^2 = 0.25 (246), p = 0.62
	**Delayed Recall**	**Score**	t = -2.08 (427), p = 0.06	t = -2.68 (463), p = 0.02	t = -0.55 (245), p = 0.58
		**Impaired Cognition**	χ^2 = 1.89 (428), p = 0.26	χ^2 = 5.99 (464), p = 0.04	χ^2 = 0.74 (246), p = 0.39
		**Score > AD score**	χ^2 = 2.18 (428), p = 0.21	χ^2 = 4.40 (464), p = 0.11	χ^2 = 0.17 (246), p = 0.68
	**Semantic Fluency**	**Score**	t = -2.68 (427), p = 0.01	t = -4.06 (463), p < 0.001	t = -0.64 (245), p = 0.52
		**Impaired Cognition**	χ^2 = 5.67 (428), p = 0.05	χ^2 = 3.86 (464), p = 0.08	χ^2 = 0.24 (246), p = 0.62
		**Score > AD score**	χ^2 = 3.12 (428), p = 0.12	χ^2 = 4.46 (464), p = 0.11	χ^2 = 0.02 (246), p = 0.88

[7] Benjamini-Hochberg procedure applied to all p-values.

Supplementary Table 3: Pearson correlations of age and plasma p-tau217 with cognitive scores.

		Pearson Correlations	
		**Age**	**Plasma P-tau217**
**Immediate Recall**	**Coefficient (SE)**	r = - 0.127 (0.037)	r = - 0.083 (0.040)
	**Statistics**[8]	t = - 3.34 (p < 0.001)	t = - 2.02 (p = 0.034)
**Delayed Recall**	**Coefficient (SE)**	r = - 0.202 (0.036)	r = - 0.113 (0.040)
	**Statistics**[8]	t = - 5.58 (p < 0.001)	t = - 2.80 (p = 0.006)
**Semantic Fluency**	**Coefficient (SE)**	r = - 0.149 (0.040)	r = - 0.167 (0.031)
	**Statistics**[8]	t = - 3.72 (p < 0.001)	t = - 5.41 (p < 0.001)

[8] T-statistics, standard errors, and p-values for the correlations were obtained with the survey-package svycor() function, with observations clustered by the twin participants' families.

Supplementary Table 4: Linear regression predictions for cognition with age, p-tau217 as covariates.

		Estimate	95 % CI	SE	T-value	P-value[9]
	**(Intercept)**	17.056	16.348, 17.764	0.360	47.325	< 0.001
**Immediate Recall**	**Age**	-0.476	-0.871, -0.080	0.201	-2.362	0.028
	**P-tau217**	-0.386	-1.055, 0.282	0.340	-1.135	0.257
	**(Intercept)**	4.523	4.142, 4.904	0.194	23.312	< 0.001
**Delayed Recall**	**Age**	-0.427	-0.618, -0.235	0.097	-4.385	< 0.001
	**P-tau217**	-0.253	-0.596, 0.089	0.174	-1.451	0.147
	**(Intercept)**	17.631	16.905, 18.358	0.370	47.675	< 0.001
**Semantic fluency**	**Age**	-0.576	-1.035, -0.117	0.234	-2.464	0.014
	**P-tau217**	-1.251	-1.916, -0.587	0.338	-3.701	< 0.001

Supplementary Table 5: Linear regression predictions for cognition with age, p-tau217, and age by p-tau217 interaction as covariates.

		Estimate	95 % CI	SE	T-value	P-value[9]
	**(Intercept)**	17.001	16.310, 17.692	0.352	48.317	< 0.001
**Immediate Recall**	**Age**	-0.148	-0.829, 0.533	0.347	-0.427	0.670
	**P-tau217**	-0.391	-1.041, 0.258	0.331	-1.183	0.383
	**Age:P-tau217**	0.319	-0.270, 0.908	0.300	1.065	0.383
	**(Intercept)**	4.515	4.141, 4.889	0.190	23.729	< 0.001
**Delayed Recall**	**Age**	-0.377	-0.743, -0.012	0.186	-2.027	0.086
	**P-tau217**	-0.254	-0.594, 0.086	0.173	-1.468	0.190
	**Age:P-tau217**	0.048	-0.259, 0.355	0.156	0.307	0.759
	**(Intercept)**	17.530	16.783, 18.276	0.380	46.122	< 0.001
**Semantic fluency**	**Age**	0.031	-0.744, 0.805	0.394	0.078	0.938
	**P-tau217**	-1.261	-1.927, -0.596	0.339	-3.724	< 0.001
	**Age:P-tau217**	0.591	-0.036, 1.217	0.319	1.853	0.086

[9] Benjamini-Hochberg procedure applied to all post-hoc p-values.

Supplementary Table 6: Number of impaired scores based on delayed recall and semantic fluency.[10]

			Number of Impaired Scores				Statistical Difference	
			0	1 (sf)[11]	1 (dr)[12]	2	χ^2-Statistic (DF)	P-value[13]
**Ashton et al.**[2]	**Dichotomic plasma p-tau217**	**Normal** (n = 427)	139	78	116	94	4.15 (1611)	0.006
		**Abnormal** (n = 265)	64	43	68	89		
	**Three-category plasma p-tau217**	**Low** (n = 407)	135	72	111	89	2.55 (3204)	0.02
		**Intermediate** (n = 146)	33	30	35	48		
		**High** (n = 138)	35	19	38	46		
**Figdore et al.**[1]	**Dichotomic plasma p-tau217**	**Normal** (n = 483)	152	89	127	115	2.47 (1613)	0.06
		**Abnormal** (n = 208)	51	32	57	68		
	**Three-category plasma p-tau217**	**Low** (n = 411)	135	74	113	89	2.44 (3204)	0.02
		**Intermediate** (n = 123)	31	23	28	41		
		**High** (n = 157)	37	24	43	53		

[10] Participant groups based on the number of impaired cognitive tests of semantic fluency (sf) and delayed recall (dr).
[11] Semantic fluency
[12] Delayed recall
[13] Benjamini-Hochberg procedure applied to all p-values.

Supplementary table 7: Post-hoc Chi-squared test of Alzheimer's disease neuropathological changes (ADNPC) prevalence by cognitive performance in delayed recall and semantic fluency.

	Chi-squared Test of ADNPC			
	**ADNPC Based on the Ashton cut-off (p-tau217 > 0.42)**		**ADNPC Based on the Figdore cut-off (p-tau217 > 0.475)**	
	**χ^2-statistic (DF)**	**P-value[14]**	**χ^2-statistic (DF)**	**P-value[14]**
**0 – 1 (sf)**	0.54 (277)	0.56	0.07 (277)	0.79
**0 – 1 (dr)**	1.30 (320)	0.38	1.62 (320)	0.32
**0 – 2**	11.02 (329)	0.01	6.10 (329)	0.08
**1 (sf) – 1 (dr)**	0.06 (275)	0.81	0.71 (275)	0.48
**1 (sf) – 2**	4.90 (267)	0.06	3.83 (267)	0.15
**1 (dr) – 2**	5.17 (316)	0.06	1.53 (316)	0.32

Supplementary Table 8: Post-hoc two-tailed t-tests of plasma p-tau217 levels by cognitive performance in delayed recall and semantic fluency.

	Two-tailed T-test on Plasma P-tau217 level	
	**T-statistic (DF)**	**P-value[14]**
**0 – 1 (sf)**	1.62 (276)	0.10
**0 – 1 (dr)**	1.20 (319)	0.23
**0 – 2**	3.39 (328)	< 0.001
**1 (sf) – 1 (dr)**	-0.29 (274)	0.77
**1 (sf) – 2**	1.77 (266)	0.08
**1 (dr) – 2**	1.99 (315)	0.05

[14] Benjamini-Hochberg procedure applied to all post-hoc p-values.

Supplementary Table 9: Demographics of individuals with biomarker and cognitive profiles indicating Alzheimer's disease based on plasma p-tau217 and two telephone-based cognitive measures.
Cognitive impairment was defined as performance below an education-adjusted cut-off on two telephone-based cognitive tests: delayed recall and semantic fluency.

	**ADNPC (Ashton) & Cognitive Impairment** (N = 89)	**Other TWINGEN Participants** (N = 602)	**Statistical Difference**[15]
**Age, Mean (SD)**	77.58 (3.61)	75.96 (4.67)	t = -3.48 (ddf = 538)
**Range**	68.46—85.31	65.42—85.68	p = 0.001
			χ2 < 0.001 (ddf = 539)
**Females, N (%)**	51 (57.3)	345 (57.3)	p = 1.00
**Education, Median (IQR)**	10 (6)	10 (6)	W = 0.71 (ddf = 538)
**Range**	6—18	6—18	p = 0.63
			χ2 = 21.01 (ddf = 539)
***APOE* ε4-carriers, N (%)**	45 (50.6)	155 (25.7)	p < 0.001
	**ADNPC (Figdore) & Cognitive Impairment** (N = 68)	**Other TWINGEN Participants** (N = 623)	
**Age, Mean (SD)**	77.25 (3.55)	76.05 (4.66)	t = -2.31 (ddf = 538)
**Range**	68.46—85.31)	65.42—85.68	p = 0.04
			χ2 = 0.06 (ddf = 539)
**Females, N (%)**	38 (55.9)	358 (57.5)	p = 0.81
**Education, Median (IQR)**	10 (6)	10 (6)	W = -0.40 (ddf = 538)
**Range**	8—18	8—18	p = 0.81
			χ2 = 21.30 (ddf = 539)
***APOE* ε4-carriers, N (%)**	37 (54.4)	163 (26.2)	p < 0.001

[15] Benjamini-Hochberg procedure applied to all post-hoc p-values.

Supplementary Material References

1. Figdore DJ, Griswold M, Bornhorst JA, et al. Optimizing cutpoints for clinical interpretation of brain amyloid status using plasma p-tau217 immunoassays. *Alzheimers Dement*. 2024;20(9):6506-6516. doi:10.1002/alz.14140

2. Ashton NJ, Brum WS, Di Molfetta G, et al. Diagnostic Accuracy of a Plasma Phosphorylated Tau 217 Immunoassay for Alzheimer Disease Pathology. *JAMA Neurol*. 2024;81(3):3. doi:10.1001/jamaneurol.2023.5319
